# Targeting STAT3 Signaling Facilitates Responsiveness of Pancreatic Cancer Cells to Chemoradiotherapy

**DOI:** 10.3390/cancers14051301

**Published:** 2022-03-03

**Authors:** Hannah Flebbe, Melanie Spitzner, Philipp Enno Marquet, Jochen Gaedcke, B. Michael Ghadimi, Stefan Rieken, Günter Schneider, Alexander O. Koenig, Marian Grade

**Affiliations:** 1 Department of General, Visceral and Pediatric Surgery, University Medical Center Goettingen, 37075 Goettingen, Germany; hannah.flebbe@med.uni-goettingen.de (H.F.); melanie.spitzner@med.uni-goettingen.de (M.S.); pmarquet@tabea-krankenhaus.de (P.E.M.); jochen.gaedcke@med.uni-goettingen.de (J.G.); mghadim@uni-goettingen.de (B.M.G.); guenter.schneider@med.uni-goettingen.de (G.S.); 2 Zentrum für Dermatochirurgie, Krankenhaus Tabea, 22587 Hamburg, Germany; 3 Department of Radiotherapy and Radiooncology, University Medical Center Goettingen, 37075 Goettingen, Germany; stefan.rieken@med.uni-goettingen.de; 4 Department of Gastroenterology, Gastrointestinal Oncology and Endocrinology, University Medical Center Goettingen, 37075 Goettingen, Germany; alexander.koenig@med.uni-goettingen.de

**Keywords:** pancreatic ductal adenocarcinoma, STAT3, preoperative therapy, chemoradiotherapy, resistance

## Abstract

**Simple Summary:**

Preoperative chemoradiotherapy (CRT) has emerged as a potential therapeutic strategy to increase the fraction of patients with pancreatic ductal adenocarcinoma (PDAC) who are candidates for surgical resection. However, treatment response is heterogeneous and ranges from complete response to progression. In this study, we uncovered that the transcription factor STAT3 mediates CRT resistance in PDAC cell lines with high IL-6/STAT3 signaling activity. If further validated, pharmacological inhibition of the IL-6/STAT3 pathway may represent a promising therapeutic strategy to increase responsiveness of PDAC to preoperative CRT.

**Abstract:**

The debate is ongoing regarding the potential role of preoperative chemoradiotherapy (CRT) for patients with pancreatic ductal adenocarcinoma (PDAC), and whether it should be reserved for borderline resectable or unresectable tumors. However, treatment response is heterogeneous, implicating the need to unveil and overcome the underlying mechanisms of resistance. Activation of the transcription factor STAT3 was recently linked to CRT resistance in other gastrointestinal cancers such as rectal and esophageal cancers, but its role in PDAC needs to be clarified. Protein expression and phosphorylation of STAT3 was determined in PDAC cell lines and connected to transcriptional activity measured by dual-luciferase reporter gene assays. Inhibition of STAT3 signaling was achieved by RNAi or the small-molecule inhibitor napabucasin. We observed a positive correlation between STAT3 signaling activity and CRT resistance. Importantly, genetical and pharmacological perturbation of the IL-6/STAT3 pathway resulted in CRT sensitization specifically in those cell lines, in which STAT3 activity was augmented by IL-6. In conclusion, our data underscore the general importance of IL-6/STAT3 signaling for CRT resistance and suggest that pathway inhibition may represents a putative treatment strategy in order to increase the fraction of patients with PDAC who are candidates for surgical approaches.

## 1. Introduction

Pancreatic ductal adenocarcinoma (PDAC) is a major devastating type of cancer with an increasing incidence [1] and is expected to become the second leading cause of cancer-related deaths in the USA [2]. While surgical resection, either alone or in combination with preoperative (neoadjuvant) and/or postoperative (adjuvant) therapeutic concepts, remains the central treatment component, many patients present with advanced stages, which are not amenable for surgical approaches [3].

Radiation therapy (RT) represents a putative treatment strategy to increase the fraction of patients who are candidates for surgery. However, it remains under exploration whether it should be reserved for borderline resectable or unresectable tumors, and whether it should be used in a preoperative or postoperative setting or sequentially [4,5,6,7,8]. Ideally, such clinical developments should be accompanied by preclinical research to increase our understanding of treatment resistance, define RT sensitizers, and identify markers to stratify RT resistant and sensitive tumors. Such efforts will ultimately help to implement precision RT for PDAC.

Signal transducer and activator of transcription 3 (STAT3) has been shown to play a crucial role in the development and progression of PDAC [9,10] and is frequently deregulated in multiple malignancies [11,12]. Interleukin-6 (IL-6) and cytokines of the IL-6 family, which control a plethora of cellular functions, modulate STAT3 signaling [13,14]. This modulation is highly context-dependent, as other signaling pathways in addition to STAT3 such as RAS/RAF/MEK/ERK, PI3K/AKT/mTOR, or NF-κB may be regulated by IL-6 [15,16,17,18]. For activation, IL-6 binds to its cytokine receptor, which uses the glycoprotein gp130 as a common signal transducer. The activated IL-6/IL-6 receptor/gp130 complex now recruits Janus kinase 2 (JAK2), which directly phosphorylates STAT3 at tyrosine 705 (Tyr705). Phosphorylated STAT3 (pSTAT3^Tyr705^) dimerizes and translocates to the nucleus, where it activates its target genes leading to proliferation, survival, and tumor invasion [11,12,13,19,20].

Previously, we have demonstrated that the IL-6/STAT3 signaling pathway plays a central role in mediating the resistance of rectal cancer to chemoradiotherapy (CRT) and that inhibition of IL-6/STAT3 signaling leads to the sensitization to CRT in vitro and in vivo [21,22].

In the present study, we therefore investigated the putative role of STAT3 in controlling CRT sensitivity in PDAC. Indeed, STAT3 is connected to treatment resistance, and the small-molecule inhibitor napabucasin re-sensitizes a priori resistant cells to CRT. Our data underscore the general importance of STAT3 for CRT resistance and point to a novel role for STAT3 as a potential target for radiosensitization in PDAC.

## 2. Materials and Methods

### 2.1. Cell Lines and Cell Culture

Seven human PDAC cell lines were obtained from the American Type Culture Collection (ATCC, Manassas, VA, USA): BxPC-3, Capan-1, Capan-2, L3.6, MIA PaCa-2, PANC-1, and PaTu8988t. The ATCC ensures authenticity of these cell lines using short tandem repeat profiling [23]. Cells were cultured in their recommended media (Invitrogen, Karlsruhe, Germany), supplemented with 2 mM L-glutamine (BioWhittaker, Verviers, Belgium) and 10% fetal bovine serum (Pan, Aidenbach, Germany). Periodically, contamination with mycoplasma was excluded using the MycoAlert^®^ Mycoplasma Detection Kit (Lonza, Cologne, Germany).

### 2.2. Western Blot Analyses

Western blot analysis was performed as previously described [21,22]. In short, 20 µg of whole-cell protein lysate was loaded and resolved on a 10% bis-tris polyacrylamide gel. Via semi-dry blotting, proteins were transferred to a polyvinylidene difluoride membrane (PVDF; GE Healthcare, Little Chalfont, UK). The membrane was incubated with antibodies and protein detection was performed using an ImageQuant LAS 4000 mini-CCD camera system (GE Healthcare, Freiburg, Germany). For visualization of pSTAT3^Tyr705^, cells were incubated with 100 ng/mL IL-6 for 10 min prior to lysis. The original Western blot images are shown in Figure S1, and experimental conditions and respective antibodies can be found in Table S2.

### 2.3. Dual Luciferase Reporter Assays

The STAT3 CignalTM Pathway Reporter Assay Kit (Qiagen; Hilden, Germany) was used for reporter transfections and the Dual-Luciferase^®^ Reporter Assay System (Promega, Madison, WI, USA) for visualization of luciferase activity. Experiments were performed as previously described according to the manufacturers’ instructions [22]. All cell lines were transfected using X-tremeGENE HP DNA Transfection Reagent (Roche, Mannheim, Germany) under serum-free conditions with reporter plasmid DNA including the *Renilla* luciferase reporter plasmid (Promega) for normalization (Table S3). After 24 h, cells were treated with 100 ng/mL IL-6 overnight, lysed by a passive lysis buffer (Promega), and *Renilla* and firefly activity was measured in a microplate reader (Mithras LB940, Berthold Technologies, Bad Wildbad, Germany). For analyses, normalized relative light units (RLU) of the STAT3-specific reporter and normalized values of the negative control reporter were divided. To assess STAT3 pathway activation, samples with and without stimulation with IL-6 were compared. Additional information about transfection conditions is presented in Table S3. All experiments were conducted as technical and biological triplicates.

### 2.4. RNAi-Mediated Silencing of STAT3

Silencing of STAT3 was performed as previously described [21,22] using Nucleofector^TM^ technology (Lonza). Briefly, cells growing in log-phase were transfected with small-interfering RNA (siRNA; Dharmacon/Thermo Fisher Scientific, Schwerte, Germany) targeting *STAT3* and a nonsilencing control siRNA (siNEG, Qiagen). Additional information about transfection conditions and siRNA sequences is presented in Table S4.

### 2.5. Radiation, Chemoradiotherapy and Colony Formation Assays

To determine the respective surviving fractions (SFs), standard colony formation assays (CFAs) were performed as previously reported [21,22]. Briefly, defined numbers of tumor cells growing in log-phase were seeded as single-cell suspensions into six-well plates (Table S5). For the CRT-setting, cells were incubated with 3 μM of 5-FU (Sigma-Aldrich, Steinheim, Germany) for 16 h followed by irradiation with single doses of 1, 2, 4, 6, and 8 Gy of X-rays (Gulmay Medical, Camberley, U.K.). Following siRNA-mediated gene silencing, RT and CRT were performed after defined cell-line-specific time points (Table S5) or one hour after treatment with the STAT3 inhibitor napabucasin. After a certain growth period (Table S5), cells were fixed with 70% ethanol, stained with Mayer’s hemalum solution (Merck KGaA, Darmstadt, Germany), and counted. Colonies with more than 50 cells were scored as survivors and plotted according to the linear quadratic model [24] as mean and standard error of the mean (s.e.m.). Calculated surviving fractions were plotted using KaleidaGraph software (Synergy Software, Reading, PA, USA). All experiments were assessed in technical and biological triplicates.

### 2.6. Cellular Viability Assays

Cellular viability assays were performed as previously described [21,22]. Briefly, cells were seeded into opaque black 96-well plates. To determine the cellular viability of siRNA-treated cells, reverse lipid-based transfections were performed (RNAiMAX, life technologies, Carlsbad, CA, USA). Following defined incubation periods, cellular viability was assessed using the CellTiter-Blue^®^ reagent (Promega) in a plate reader (VICTOR^TM^ X4, Perkin Elmer, Waltham, MA, USA). All experiments were conducted as technical and biological triplicates.

### 2.7. Statistical Analysis

All experiments, except for Western blot analyses, were performed as technical and biological triplicates. Dual luciferase and cell viability assays were analyzed by an unpaired two-tailed Student’s *t*-test in Microsoft Excel (version 2016), and visualized in Grapher software (version 8.2.460, Golden Software, Golden, CO, USA). Correlations were calculated with Pearson’s correlation in Microsoft Excel (version 2016). Statistical analyses of irradiation experiments were performed with two-way analysis of variance (ANOVA) to calculate significant differences between control and treatment groups using Microsoft Excel (version 2016, Add-in “Data Analysis”, Microsoft Corporation, Redmond, WA, USA). *p*-values < 0.05 were defined as significant.

## 3. Results

### 3.1. STAT3 Expression, Phosphorylation of STAT3 and STAT3 Transcriptional Activity

**Figure 1:**
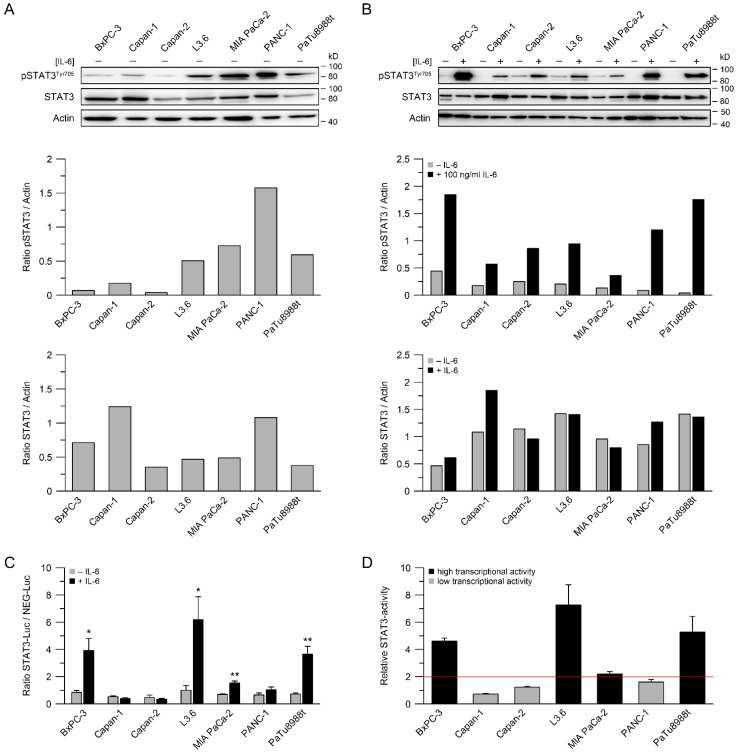
STAT3 expression and phosphorylation, and STAT3 transcriptional activity. (**A**) Seven pancreatic adenocarcinoma cell lines were analyzed for expression of STAT3 and levels of phosphorylated STAT3 (pSTAT3^Tyr705^) by immunoblotting; (**B**) comparison of STAT3 and pSTAT3^Tyr705^ protein levels after stimulation with interleukin-6 (IL-6; 100 ng/mL); (**C**,**D**) analysis of STAT3 transcriptional activity with and without stimulation with IL-6, displayed as STAT3/NEG-luciferase ratio (**C**), or relative STAT3 transcriptional activity (**D**), measured by dual luciferase reporter assays. Data are presented as mean ± s.e.m. from at least *n* = 3 independent biological replicates. * *p* < 0.05, ** *p* < 0.01, unpaired two-sample Student’s *t*-test. For *p*-values, see Table S1.

We have recently shown that increased STAT3 activity correlates with chemoradiotherapy resistance in rectal and esophageal cancer cells, and that inhibition of the IL-6/STAT3 pathway results in sensitization to CRT [21,22]. To expand our analyses to PDAC, we first determined the expression and phosphorylation of STAT3 by Western blotting in seven human PDAC cell lines under nonstimulated conditions. Although protein levels differed, STAT3 was expressed in all seven cell lines (Figure 1A). In particular, L3.6, MIA PaCa-2, PANC-1, and PaTu8988t showed high levels of the activated Tyr705-phosphorylated form of STAT3 (Figure 1A). Since phosphorylation of STAT3 can be induced by external stimuli, especially by the cytokine IL-6, we measured pSTAT3^Tyr705^ levels in response to IL-6. In all cell lines, IL-6 induced phosphorylation of STAT3, while expression levels of unphosphorylated STAT3 remained unchanged (Figure 1B). The ratios of phosphorylated and total STAT3, either with or without stimulation with IL-6, are depicted for all cell lines in Figure S2. In addition to the protein expression and phosphorylation, we determined STAT3 transcriptional activity using a luciferase reporter assay. Stimulation with IL-6 mediated a significant increase in transcriptional activity in four of seven PDAC cell lines, i.e., BxPC-3, L3.6, MIA PaCa-2, and PaTu8988t (Figure 1C,D). These cell lines were therefore considered as STAT3-signaling-active.

### 3.2. STAT3 Transcriptional Activity Correlates with (Chemo-) Radiotherapy Resistance

**Figure 2:**
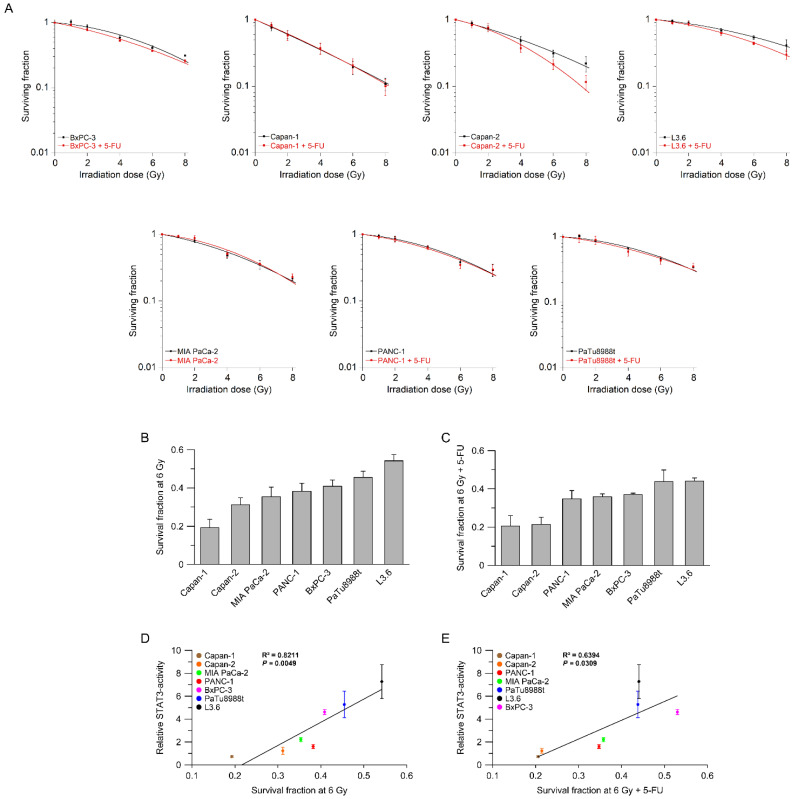
Transcriptional activity of IL-6/STAT3 signaling correlates with sensitivity of pancreatic adenocarcinoma cell lines to (chemo)radiotherapy. (**A**) Cell lines were assessed by colony formation assays (CFAs) to measure their survival following irradiation (RT, black lines), or irradiation in the presence of 5-FU (CRT, red lines); (**B**) comparison of survival fraction at 6 Gy (SF6) after irradiation alone or (**C**) 5-FU-based chemoradiotherapy; (**D**,**E**) SF6 (RT, **D**; CRT, **E**) correlated with STAT3 transcriptional activity. Data are presented as mean ± s.e.m. from at least *n* = 3 independent biological replicates. Pearson’s correlation.

Next, to assess whether STAT3 activity correlates with RT/CRT responsiveness, we determined the sensitivity of these cell lines using a standard colony formation assay (CFA). We used single irradiation doses from 1 to 8 Gy, with or without the chemotherapeutic agent 5-FU. Interestingly, five cell lines showed no difference in response when RT and CRT were compared, suggesting that 5-FU mediated no additional radiosensitization effect (Figure 2A), additionally supporting the need to establish predictive biomarkers. Only Capan-2 and L3.6 seemed to benefit from the addition of 5-FU (Figure 2A). To compare the individual response rates of these cell lines, we plotted their corresponding surviving fractions at 6 Gy for RT (Figure 2B) and CRT (Figure 2C). We observed heterogeneous RT/CRT response profiles, which recapitulates clinical reality. Importantly, we determined a significant positive correlation between STAT3 transcriptional activity and treatment response (Figure 2D,E), indicating a potential role of STAT3 in mediating RT/CRT resistance in PDAC cell lines.

### 3.3. STAT3 Mediates Resistance to Radiotherapy and Chemoradiotherapy

**Figure 3:**
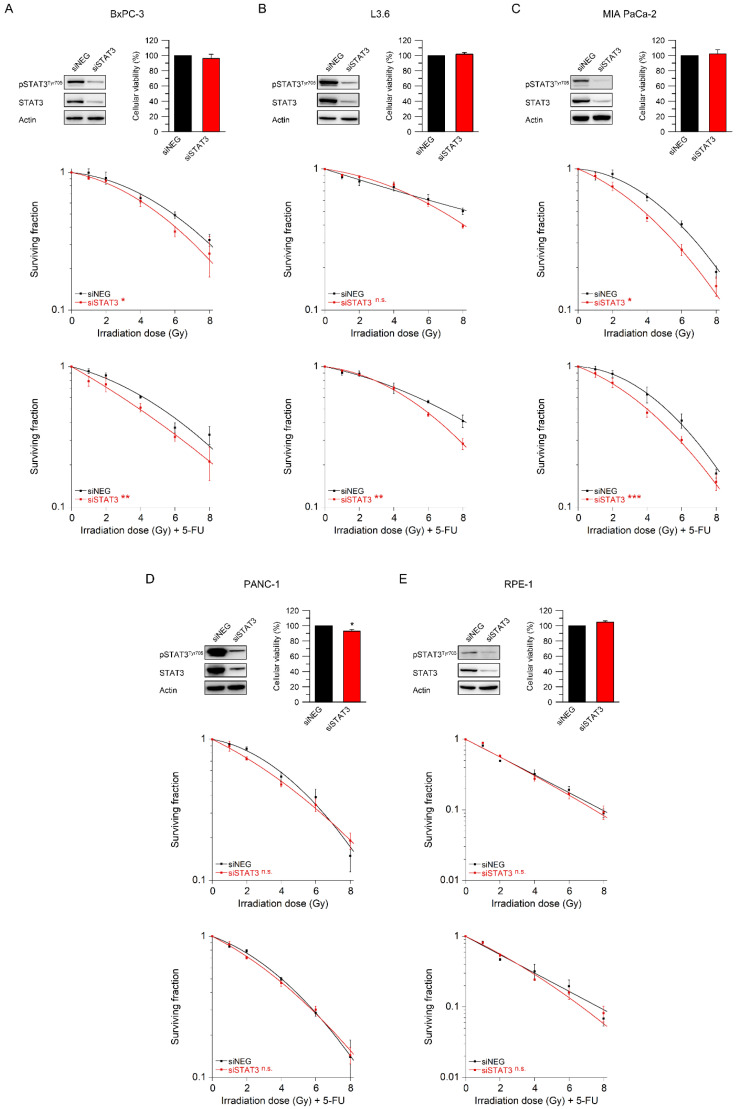
Sensitization to radiotherapy and chemoradiotherapy depends on IL-6/STAT3 pathway activity. (**A**–**E**) STAT3-active cell lines BxPC-3 (**A**), L3.6 (**B**), and MIA PaCa-2 (**C**); the STAT3-inactive cell line PANC-1 (**D**); and the normal retinal epithelial cell line RPE-1 (**E**) were transfected with control siRNA (siNEG) or an siRNA pool targeting STAT3, and, after stimulation with 100 ng/mL IL-6, subjected to immunoblot analyses (upper left) or cellular viability assays (upper right). Following siRNA-mediated STAT3 silencing, cells were monitored for CFA survival after irradiation alone (middle panel) or RT in the presence of 5-FU (CRT) (lower graph). Data are presented as mean ± s.e.m. from at least 3 independent biological replicates. * *p* < 0.05, ** *p* < 0.01, *** *p* < 0.001, unpaired two-sample Student’s *t*-test or two-way analysis of variance (ANOVA). For *p*-values, see Table S1.

To functionally assess whether STAT3 confers CRT resistance, we reduced STAT3 expression with RNAi. We selected three cell lines with high STAT3 activity (BxPC-3, L3.6, and MIA PaCa-2), one cell line with low STAT3activity (PANC-1), and the normal epithelial cell line RPE-1. Successful silencing of STAT3 was validated by Western blot analysis (Figure 3A–E, upper left panel). There was no dramatic effect of the siRNA transfection on cellular viability in the absence of RT/CRT (Figure 3A–E, upper right panel).

Next, we assessed the influence of RNAi-mediated STAT3 inhibition on sensitivity to RT and CRT via standard colony formation assays. As shown in Figure 3A–C (middle and lower panel), RNAi-mediated silencing of STAT3 significantly re-sensitized BxPC-3 and MIA PaCa-2 cells to both radiotherapy alone and CRT, while, in L3.6 cells, we observed a significant sensitization effect in the presence of CRT. In clear contrast, there was no sensitization to RT or CRT in PANC-1 cells, which are characterized by low STAT3 activity (Figure 3D, middle and lower panel). Similarly, there was no effect in nontumorigenic RPE-1 cells (Figure 3E, middle and lower panel). To summarize, these data suggest that STAT3 mediates treatment resistance in PDAC cell lines that are IL-6/STAT3-active.

### 3.4. The STAT3 Inhibitor Napabucasin Sensitizes to RT and CRT

**Figure 4:**
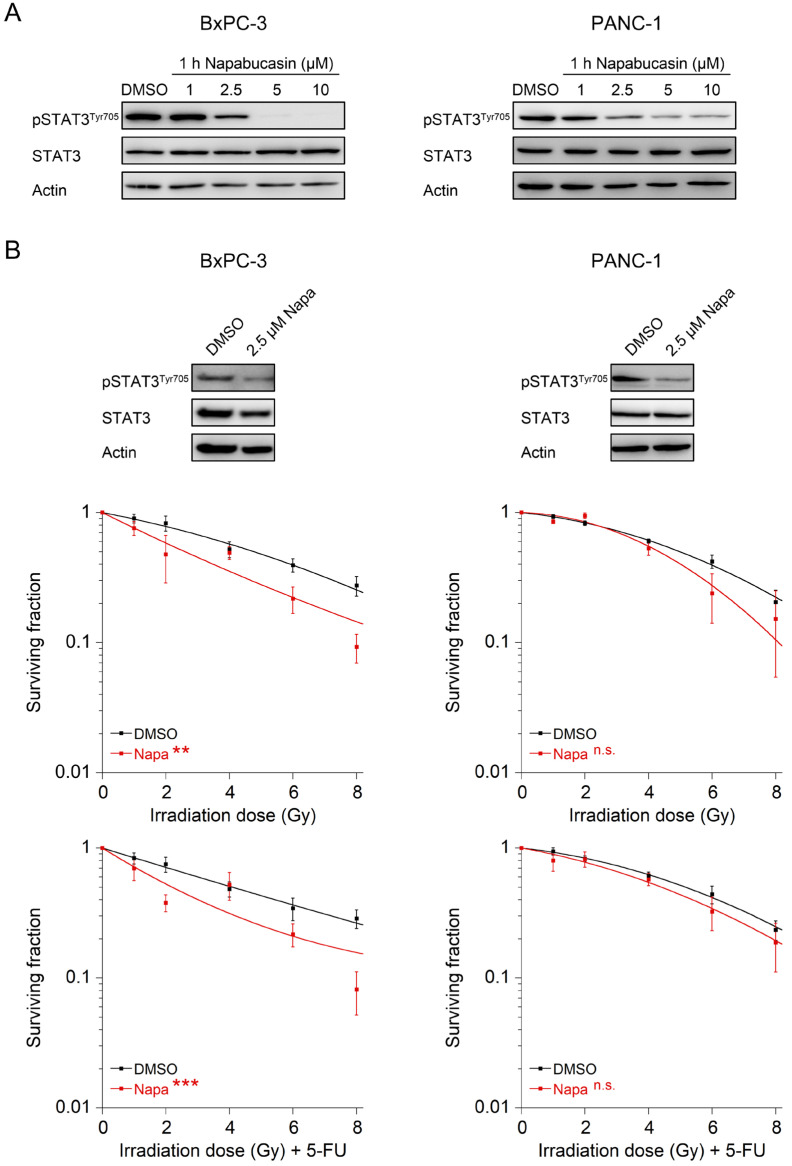
Pharmacological inhibition of STAT3 signaling with napabucasin induces re-sensitization to irradiation and chemoradiotherapy. (**A**) Establishment of effective napabucasin (Napa) concentrations in BxPC-3 (left panel) and PANC-1 cells (right panel); (**B**) BxPC-3 cells (left panels) and PANC-1 cells (right panels) were left untreated (DMSO) or treated with the STAT3 inhibitor napabucasin for 1 h. Depletion of pSTAT3^Tyr705^ was monitored by Western blot analysis after stimulation with 100 ng/mL IL-6 (upper panel), and cells were subjected to CFA survival after RT (upper graphs) or CRT (lower graphs). Data are presented as mean ± s.e.m. from at least 3 independent biological replicates. ** *p* < 0.01, *** *p* < 0.001, two-way analysis of variance (ANOVA). For *p*-values, see Table S1.

Finally, to evaluate whether this may offer a translational treatment option, i.e., whether STAT3 inhibition can be explored clinically, we used napabucasin, a small-molecule STAT3 inhibitor (BBI608 [25]) which has already been tested in different phase III trials (NCT03721744 and NCT02993731 [26]). For these experiments, we decided to compare a STAT3-active (BxPC-3) and a STAT3-inactive cell line (PANC-1). In both cell lines, treatment with increasing concentrations of napabucasin resulted in reduced levels of pSTAT3^Tyr705^ at a concentration of 2.5 µM after treatment for 1 h (Figure 4A), which was therefore chosen as the concentration for further experiments. Of note, treatment with napabucasin significantly re-sensitized the STAT3-active cell line BxPC-3 to both RT and CRT (Figure 4B, left panels). However, there was no significant effect in the STAT3-inactive cell line PANC-1 (Figure 4B, right panels). These data support the conclusion that PDAC with high STAT3 transcriptional activity may benefit from napabucasin in an RT and CRT regimen.

## 4. Discussion

There is an ongoing and controversial debate on the potential role of preoperative treatment of PDAC [6,7,8]. In theory, preoperative treatment offers several advantages: First, it may help to select patients who are candidates for surgery, because tumors that progress during treatment may represent very aggressive subtypes that might not benefit from surgical approaches. Second, administering therapy upfront ensures that patients actually receive systemic treatment, which is postponed in the adjuvant setting in a large proportion of patients due to postoperative complications [27,28,29]. Finally, preoperative treatment may increase the likelihood of an R0 resection, which represents one of the most important prognostic factors [6].

In this context, it also remains unclear whether preoperative treatment should consist of chemotherapy (preferably FOLFIRINOX), (chemo-)radiotherapy, or a (sequential) combination thereof, and whether preoperative treatment should be reserved for borderline resectable tumors, who are at high risk of an R1 resection, or unresectable tumors [8,30,31,32]. Versteijne et al. recently published the results of the randomized PREOPANC-1 trial, in which patients with resectable or borderline-resectable PDAC were either treated with gemcitabine-based chemoradiotherapy followed by surgical resection or with immediate surgery [31]. The authors reported higher R0 resection and better disease-free survival rates after preoperative chemoradiotherapy, while median overall survival was not significantly different between both treatment arms. However, when subgroup analyses of patients with borderline-resectable tumors were performed, they also observed significantly improved overall survival rates after preoperative chemoradiotherapy (median survival 17.6 vs. 13.2 months, *p* = 0.029). Regarding patients with initially unresectable, locally advanced PDAC, Fietkau et al. recently reported in their interim analysis of the randomized CONKO-007 trial, in which patients without tumor progression after induction chemotherapy (gemcitabine or FOLFIRINOX) either received chemotherapy alone or concurrent chemoradiotherapy, that even in this stage of the disease, an R0 resection may be achieved in up to 20% of patients [33].

While these data point to an important role of preoperative treatment strategies for PDAC, they also highlight two central aspects: First, treatment response is not uniform, and ranges from progression to complete histopathological response. Second, clinicians are faced with the dilemma that the individual patient’s response to it is currently not predictable. Accordingly, there is a strong clinical need to identify the genetic mechanisms and pathways underlying treatment resistance, because this may lead to the identification of effective strategies to overcome it. As a consequence, it may increase the fraction of tumors that respond to preoperative treatment, ultimately resulting in higher rates of R0 resections, which remains one of the most important prognostic factors.

Here, we show that IL-6/STAT3 signaling mediates CRT resistance and that pathway inhibition re-sensitizes PDAC cells with high STAT3 transcriptional activity to CRT. In this context, our data support the growing body of evidence that STAT3 may represent a potential therapeutic target to overcome CRT resistance in various tumor entities [13,34]. Rectal cancer is a prime example. We recently demonstrated that perturbation of STAT3 signaling using either RNAi or the STAT3 inhibitor napabucasin re-sensitized treatment-refractory rectal cancer cells to CRT and abolished tumor growth in vivo [22]. In addition, re-expression of wild-type STAT3 in a STAT3-deficient CRC cell line increased CFA survival [22]. Similar results were recently reported by Nagaraju et al., who observed a sensitization of microsatellite-instable colon cancer cells to CRT following treatment with napabucasin [35]. Ebbing et al. previously published that IL-6 mediates resistance of esophageal adenocarcinoma cells to CRT [36]. Primary tumor cells were treated with paclitaxel or carboplatin-based CRT and subsequently incubated with IL-6-containing supernatants derived from cancer-associated fibroblasts (CAFs), which induced treatment resistance. This effect could be reverted with an IL-6-neutralizing antibody [36]. Regarding PDAC, Wu et al. demonstrated that treatment with the synthetic curcumin analog FLLL32 mediated STAT3 pathway inhibition and increased RT response in vitro and in vivo [37]. In contrast to our data, the authors observed that treatment of PANC-1, a cell line with a low transcriptional STAT3 activity in our study (Figure 1C,D), increased sensitivity to RT in clonogenic survival assays [37]. In our study, however, there was no sensitization of PANC-1 cells to CRT following RNAi-mediated silencing of STAT3 or treatment with napabucasin (Figure 3D and Figure 4). It remains unclear whether this discrepancy is due to the off-target effects of FLLL32, or due to different RT-sensitivities of PANC-1—very high in the study by Wu et al. [37], but rather low in our study (Figure 2A).

As discussed above, there is growing evidence that STAT3 inhibition may represent a potential therapeutic concept for tumors with high STAT3 signaling activity. To translate these findings into the clinic, effective pathway inhibitors are needed. Napabucasin has already been tested in a phase III clinical trial (NCT01830621) for advanced CRC [26]. In this multicenter study, patients with chemotherapy-refractory CRC either received placebo or napabucasin. While there was no difference in overall survival between the groups for the entire patient population, only 22% of tumors were pSTAT3-positive. Importantly, in a subgroup analysis of pSTAT3-positive patients, an overall survival benefit was observed. Therefore, stratification for the napabucasin-sensitive group is necessary. Similarly, napabucasin was tested in the CanStem111P trial, a phase III study in which nab-paclitaxel and gemcitabine were compared, either alone or in combination with napabucasin, as first-line treatment of patients with metastatic PDAC [38]. Overall and disease-free survival were not improved in the napabucasin group [39]. Importantly and in contrast to CRC, the pSTAT3-positive subgroup did not benefit from the addition of napabucasin. Whether pSTAT3 serves as a biomarker for napabucasin-dependent radiosensitization or whether a more direct measure of dynamic STAT3 transcriptional activity is needed for stratification awaits further clarification.

Mechanistically, it remains to be elucidated how STAT3 signaling mediates CRT resistance and which downstream pathways and targets are affected. Regarding napabucasin, evidence suggests that its sensitizing effect in combination with RT may be explained by the ability of both treatments to alter redox homeostasis, which may lead to an increased generation of reactive oxygen species (ROS) [35,40,41]. In addition to other effects, irradiation causes the production of ROS from ionized water molecules [42], which induces DNA damage, protein misfolding and misfunction, as well as cell death [43,44]. Furthermore, decreased ROS levels in cancer have recently been associated with treatment resistance and cancer stem cells, while increased ROS levels resulted in sensitization to radiation in different tumor entities [45,46,47]. Although inhibitors of the STAT3 pathway have been connected to increased intracellular ROS levels [48,49,50,51,52], the effects of the combination of napabucasin with CRT in context of PDAC toward ROS homeostasis and ROS-dependent cell death remain to be determined.

## 5. Conclusions

Our results indicate that for a highly selected subgroup of patients with PDAC with high IL-6/STAT3 signaling activity, pharmacological pathway inhibition may represent a promising therapeutic strategy to enhance responsiveness to CRT. If further validated, STAT3 inhibition may be tested in preoperative treatment concepts.

## Data Availability

Data can be found in this publication or requested from the corresponding author.

## Supplementary Materials

The following supporting information can be downloaded at: https://www.mdpi.com/article/10.3390/cancers14051301/s1, Figure S1: Original immunoblotting images presented in the paper, Figure S2: Calculated ratios of protein levels of phosphorylated STAT3 and total STAT3, Table S1: *p*-values of irradiation experiments and cellular viability assays, Table S2: Antibodies for Western blot analyses, Table S3: Experimental conditions for dual luciferase reporter assays and cellular viability assays, Table S4: siRNA sequences, Table S5: Experimental conditions for colony formation assays.
