# Supplementary material for: Targeting STAT3 Signaling Facilitates Responsiveness of Pancreatic Cancer Cells to Chemoradiotherapy
Source: Cancers (Basel). 2022 Mar 3;14(5):1301. doi: 10.3390/cancers14051301 (PMC8908974; doi:10.3390/cancers14051301)
Supplement: Supplementary file 1 [file cancers-14-01301-s001.zip › cancers-1600113-supplementary.pdf]

cancers MDPI

Supplementary Materials

Inhibition of STAT3 sensitizes pancreatic adenocarcinoma cells to chemotherapy and radiation

Hannah Flebbe, Melanie Spitzner, Philipp Enno Marquet, Jochen Gaedcke, B. Michael Ghadimi, Stefan Rieken, Günter Schneider, Alexander O. Koenig and Marian Grade

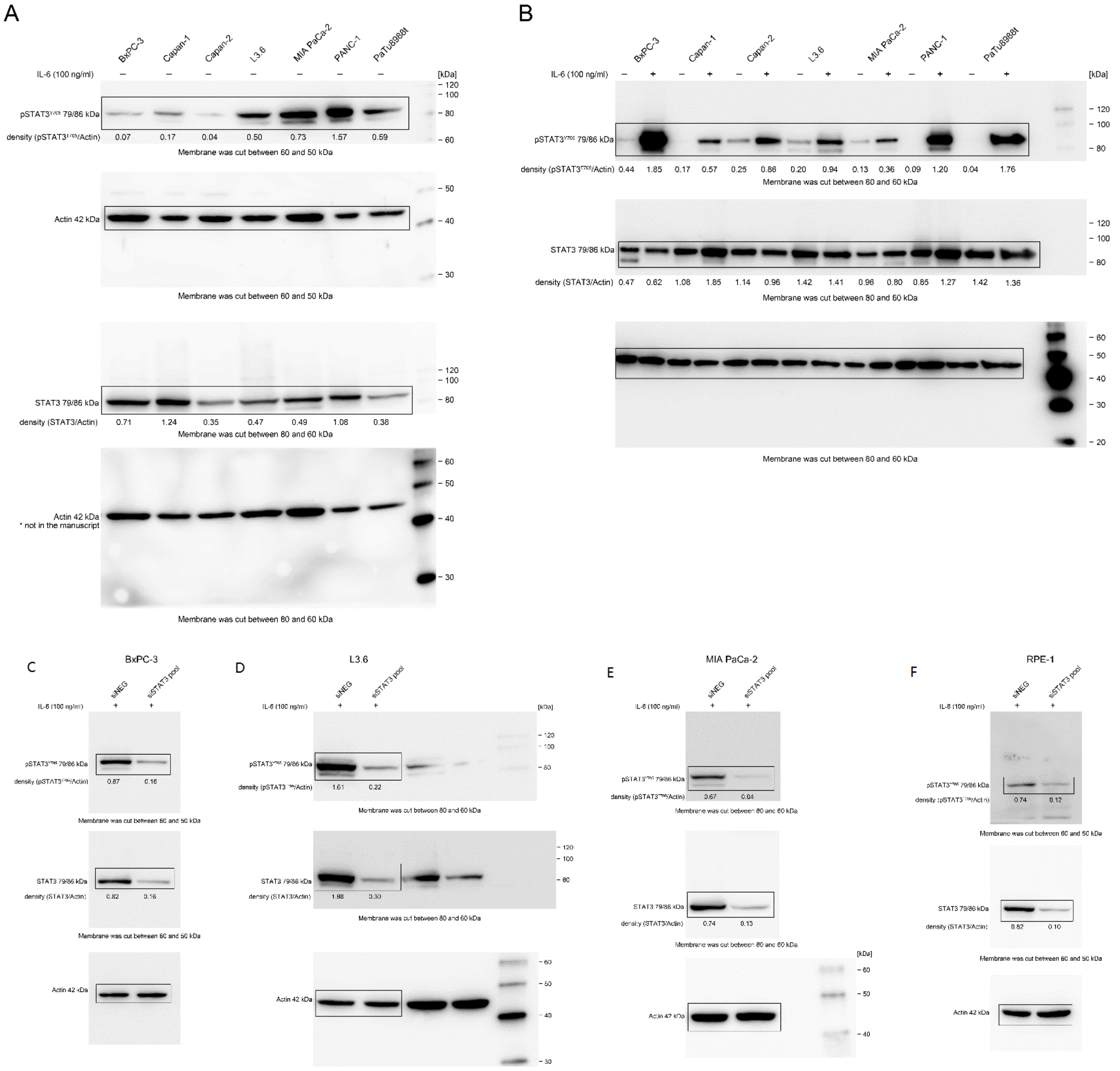

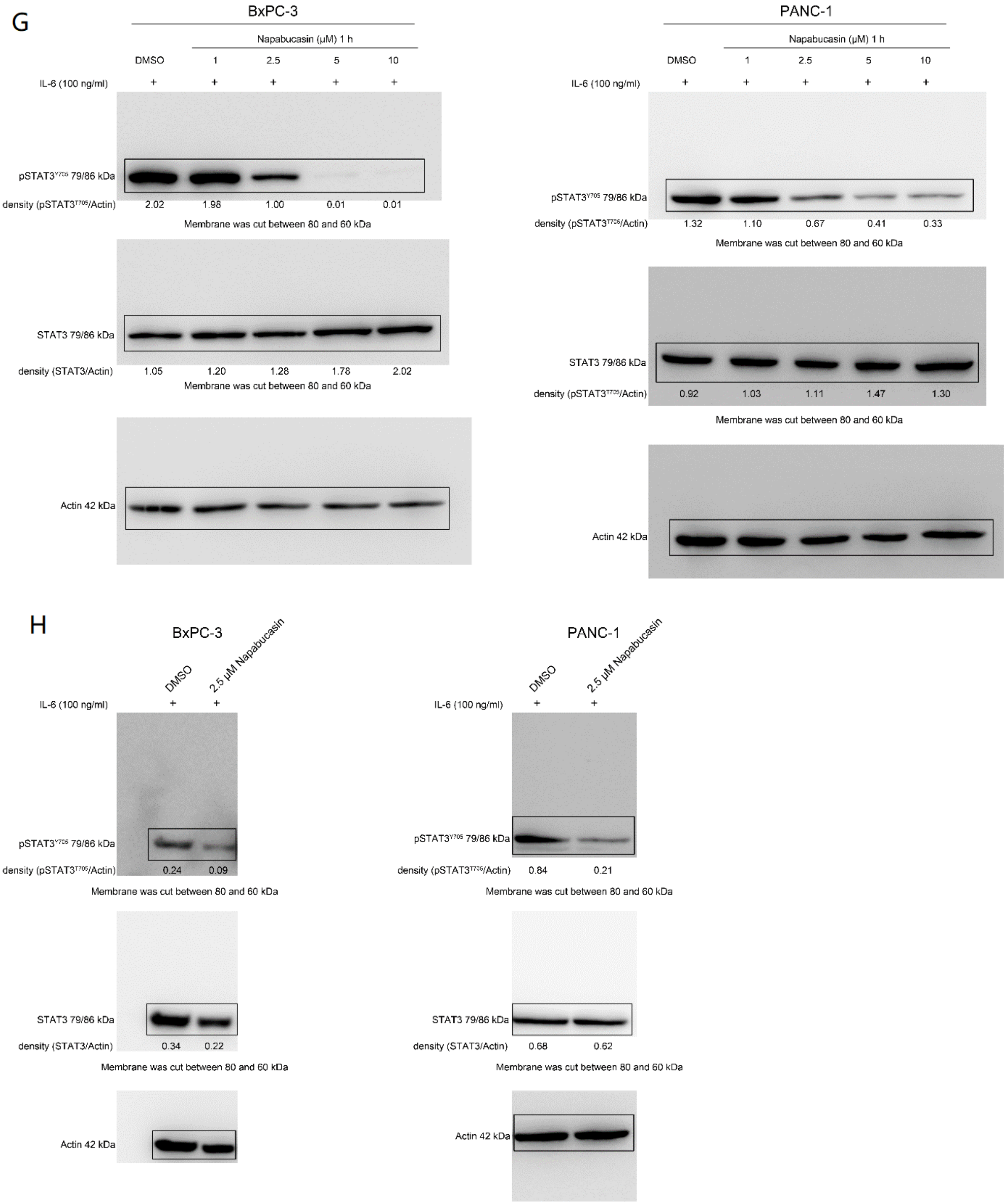

Figure S1. Original immunoblotting images. (**A,B**) For Figure 1A,B in main text. (**C-F**) For Figure 3A-C,E in main text. (**G,H**) For Figure 4A,B in main text.

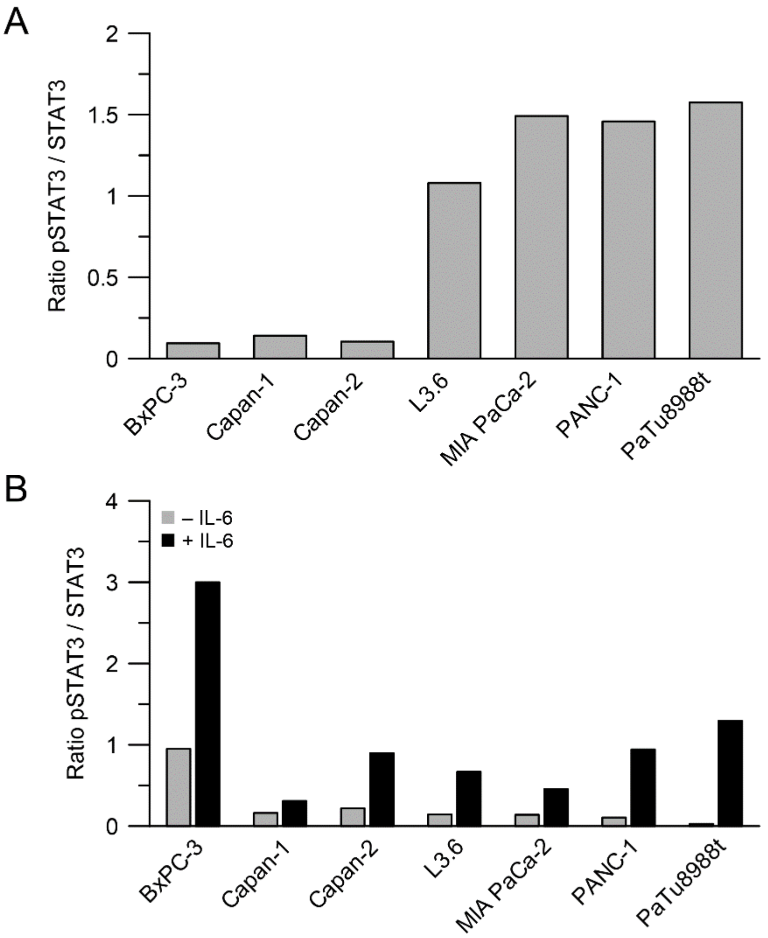

Figure S2. Calculated ratios of protein levels of phosphorylated STAT3 and total STAT3. (**A**) Seven pancreatic adenocarcinoma cell lines were analyzed for expression levels of total STAT3, and levels of phosphorylated STAT3 ($pSTAT3^{Tyr705}$) by immunoblotting; ratios of $pSTAT3^{Tyr705}$ to STAT3 are depicted; (**B**) Western blots of total STAT3 and $pSTAT3^{Tyr705}$ before and after stimulation with interleukin-6 (IL-6; 100 ng/ml); ratios of $pSTAT3^{Tyr705}$ to STAT3 are depicted.

Table S1. *P*-values of irradiation experiments and viability assays.

Cell Line	Treatment	*P*-Value DLR*	*P*-Value CTB*	*P*-Value RT+	*P*-Value CRT+
BxPC-3	Ctrl. vs. IL-6	0.0255	n.a.	n.a.	n.a.
Capan-1	Ctrl. vs. IL-6	0.1680	n.a.	n.a.	n.a.
Capan-2	Ctrl. vs. IL-6	0.6894	n.a.	n.a.	n.a.
L3.6	Ctrl. vs. IL-6	0.0173	n.a.	n.a.	n.a.
MIA PaCa-2	Ctrl. vs. IL-6	0.0057	n.a.	n.a.	n.a.
PANC-1	Ctrl. vs. IL-6	0.2081	n.a.	n.a.	n.a.
PaTu8988t	Ctrl. vs. IL-6	0.0025	n.a.	n.a.	n.a.
BxPC-3	siNEG vs. siSTAT3	n.a.	0.5179	0.0365	0.0073
L3.6	siNEG vs. siSTAT3	n.a.	0.4843	0.1028	0.0011
MIA PaCa-2	siNEG vs. siSTAT3	n.a.	0.7017	0.0125	0.0007
PANC-1	siNEG vs. siSTAT3	n.a.	0.0378	0.2343	0.8467
RPE-1	siNEG vs. siSTAT3	n.a.	0.0641	0.0814	0.735
BxPC-3	Ctrl. vs. Napabucasin	n.a.	n.a.	0.001	9.56E-04
PANC-1	Ctrl. vs. Napabucasin	n.a.	n.a.	0.157	0.1867

* unpaired two-sample Students t-test, + two-way analysis of variance (ANOVA), DLR = dual luciferase reporter assay, CTB = CellTiter-Blue®, RT = radiotherapy, CRT = chemoradiotherapy.

Table S2. Antibodies for Western blot analyses.

Protein	Clone	Host	Size (kD)	Dilution	Incubation time	Company	Catalogue number
Actin	Polyclonal	Rabbit	42	1:10,000	Over night	Sigma-Aldrich	A2066
pSTAT3Tyr705	D3A7	Rabbit	79/86	1:1,500	Over night	Cell Signaling	9145
STAT3	Polyclonal	Rabbit	79/86	1:4,000	Over night	Cell Signaling	12640
Anti-rabbit IgG-HRP	n.a.	Goat	n.a.	1:30,000	2 h	Acris	R1364HRP

kD = kilo Dalton, n.a. = not applicable, IgG = immunoglobulin G, HRP = horseradish peroxidase.

Table S3. Experimental conditions for dual luciferase reporter assays and cellular viability assays.

Cell line	Treatment	DLR			CTB		
		Cell number	Transfection reagent	DNA amount	Cell number	Transfection reagent	siRNA / lipid
BxPC-3	CignalTM Pathway Reporter	100,000	X-tremeGENE HP	1 µg (Firefly) 50ng (Renilla)	n.a.	n.a.	n.a.
	siRNA	n.a.	n.a.	n.a.	2,000	RNAiMAX	10 nM / 0.1 µl
Capan-1	CignalTM Pathway Reporter	200,000	X-tremeGENE HP	1 µg (Firefly) 50ng (Renilla)	n.a.	n.a.	n.a.
	siRNA	n.a.	n.a.	n.a.	2,000	RNAiMAX	10 nM / 0.1 µl
Capan-2	CignalTM Pathway Reporter	200,000	X-tremeGENE HP	1 µg (Firefly) 50ng (Renilla)	n.a.	n.a.	n.a.
	siRNA	n.a.	n.a.	n.a.	3,000	RNAiMAX	10 nM / 0.3 µl
L3.6	CignalTM Pathway Reporter	150,000	X-tremeGENE HP	1 µg (Firefly) 50ng (Renilla)	n.a.	n.a.	n.a.
	siRNA	n.a.	n.a.	n.a.	2,000	RNAiMAX	10 nM / 0.1 µl
MIA PaCa-2	CignalTM Pathway Reporter	150,000	X-tremeGENE HP	1 µg (Firefly) 50ng (Renilla)	n.a.	n.a.	n.a.
	siRNA	n.a.	n.a.	n.a.	2,000	RNAiMAX	10 nM / 0.7 µl
PANC-1	CignalTM Pathway Reporter	150,000	X-tremeGENE HP	1 µg (Firefly) 50ng (Renilla)	n.a.	n.a.	n.a.
	siRNA	n.a.	n.a.	n.a.	3,000	RNAiMAX	10 nM / 0.6 µl
PaTu8988t	CignalTM Pathway Reporter	75,000	X-tremeGENE HP	1 µg (Firefly) 50ng (Renilla)	n.a.	n.a.	n.a.
	siRNA	n.a.	n.a.	n.a.	2,000	RNAiMAX	10 nM / 0.1 µl
RPE-1	CignalTM Pathway Reporter	n.a.	n.a.	n.a.	n.a.	n.a.	n.a.
	siRNA	n.a.	n.a.	n.a.	1,000	siLentFect	10 nM / 0.1 µl

DLR = dual luciferase assay, CTB = CellTiter-Blue® Cell Viability Assay, 5-FU = 5-fluorouracil, n.a. = not applicable, S33Y = mutated β-catenin-S33Y.

Table S4. siRNA sequences.

Gene	Target sequence	Size (bp)	Accession number	Company	Catalogue number
Negative control (AllStarsNEG)	CAGGGTATCGACGATTACAAA	21	n.a.	Qiagen	SI03650318
STAT3-pool	GAGAUUGACCAGCAGUAUA	19	NM_003150	Qiagen	L-003544-00
	CAACAUGUCAUUUGCUGAA	19	NM_003150	Dharmacon	L-003544-00
	CCAACAAUCCCAAGAAUGU	19	NM_003150	Dharmacon	L-003544-00
	CAACAGAUUGCCUGCAUUG	19	NM_00315	Dharmacon	L-003544-00

bp = base pair, n.a. = not applicable.

Table S5. Experimental conditions for colony formation assays.

Cell line	Treatment	Transfection method time pre-irradiation	Cell number 0, 1, 2 Gy	Cell number 4 Gy	Cell number 6 Gy	Cell number 8 Gy	Growth period (days)
BxPC-3	siRNA (16 nM)	Amaxa, 48 hours	850	1,700	2,550	3,400	9
	DMSO/Napa (2.5 μM)	1 hour	850	1,700	2,550	3,400	13
L3.6	siRNA (16 nM)	Amaxa, 48 hours	150	300	450	600	8
MIA PaCa-2	siRNA (16 nM)	Amaxa, 72 hours	400	800	1,200	1,600	6
PANC-1	siRNA (16 nM)	Amaxa, 72 hours	400	800	1,200	1,600	7
	DMSO/Napa (2.5 μM)	1 hour	400	800	1,200	1,600	
RPE-1	siRNA (16 nM)	Amaxa, 72 hours	200	600	800	1,000	7

CFA = colony formation assay, DMSO = dimethyl sulfoxide, n.a. = not applicable.

Copyright: © 2022 by the authors. Licensee MDPI, Basel, Switzerland. This article is an open access article distributed under the terms and conditions of the Creative Commons Attribution (CC BY) license (https://creativecommons.org/licenses/by/4.0/).(https://creativecommons.org/licenses/by/4.0/).
